# Mitochondrial and Epigenetic Drivers of Skeletal Muscle Dysfunction in Chronic Obstructive Pulmonary Disease

**DOI:** 10.3390/antiox15070837

**Published:** 2026-07-02

**Authors:** Qian Gao, Yayun Mao, Shu Xie, Wendi Wang, Jun Xia, Weibing Wu

**Affiliations:** School of Exercise and Health, Shanghai University of Sport, Shanghai 200438, China; 2411517001@sus.edu.cn (Q.G.); maoyayun@sus.edu.cn (Y.M.); xieshu@sus.edu.cn (S.X.); 2511517004@sus.edu.cn (W.W.)

**Keywords:** chronic obstructive pulmonary disease, skeletal muscle dysfunction, mitochondrial dysfunction, oxidative stress, epigenetic regulation, targeted therapy

## Abstract

Skeletal muscle dysfunction (SMD) is a critical extrapulmonary comorbidity in chronic obstructive pulmonary disease (COPD), contributing to exercise intolerance, poor quality of life, and increased mortality. Building upon and extending the disuse model, this review synthesizes evidence establishing COPD-induced SMD as a distinct myopathy with intrinsic disease drivers. Its pathophysiology is driven by a self-reinforcing network: mitochondrial energetic crisis featuring bioenergetic failure and dysregulated dynamics, chronic oxidative stress and inflammation fueling catabolic drive via ubiquitin–proteasome system activation, and epigenetic dysregulation through alterations in key histone deacetylases (HDACs) and microRNA expression, which collectively orchestrate a pro-atrophic phenotype. We further explore how these molecular insights are translating into novel diagnostic tools, including circulating biomarkers like myomiRs and C-terminal agrin fragment, and imaging techniques such as shear wave elastography. Although exercise training remains the cornerstone of management, its limited efficacy underscores the need for adjunctive and targeted therapies. We discuss promising strategies from pharmacological and nutritional support to emerging agents targeting specific pathways, including the IL-36 receptor, lipoprotein-associated phospholipase A2, aryl hydrocarbon receptor, and mitsugumin 53. Effective management of COPD-related SMD will hinge on a precision medicine framework, leveraging biomarker-guided stratification to deploy personalized combinatorial interventions aimed at preserving muscle mass and function.

## 1. Introduction

Chronic obstructive pulmonary disease (COPD) represents a significant and growing global health challenge, currently ranking as the third leading cause of death worldwide. It is characterized by persistent respiratory symptoms and airflow limitation due to airway and/or alveolar abnormalities, typically caused by significant exposure to noxious particles or gases, most commonly cigarette smoke (CS) [1,2,3]. Traditionally, clinical management and research have predominantly focused on the pulmonary manifestations of the disease. However, it is now widely recognized that COPD is a complex systemic disorder, accompanied by a range of extrapulmonary comorbidities that profoundly influence disease severity, patient quality of life, and overall prognosis [4,5].

Among these systemic effects, skeletal muscle dysfunction (SMD) stands out as a pivotal yet frequently overlooked determinant of clinical outcomes [6,7]. COPD-related SMD presents as a syndrome characterized by muscle atrophy, dynapenia, and premature fatigue, leading to severely impaired exercise capacity, increased disability, and higher mortality rates [8,9]. For decades, the prevailing hypothesis attributed SMD primarily to disuse atrophy, a logical consequence of reduced physical activity driven by dyspnea and deconditioning. While inactivity undoubtedly plays a contributory role, emerging evidence compellingly argues that this view is insufficient. Seminal studies have demonstrated that even when matched for physical activity levels, patients with COPD exhibit distinct muscular impairments that cannot be fully explained by disuse alone [10,11]. Furthermore, interventional studies reveal a critical clinical paradox: although exercise training remains the cornerstone of pulmonary rehabilitation and can improve muscle strength and functional capacity, it often fails to fully reverse the underlying molecular abnormalities in COPD muscle, unlike in healthy trained individuals [10,12,13]. This fundamental observation underscores that intrinsic disease-specific pathophysiological processes are at play.

The current understanding points towards a multifactorial pathology where mitochondrial dysfunction emerges as a core mechanism, intricately linked with other pathways. Deficiencies in oxidative phosphorylation (OXPHOS), excessive reactive oxygen species (ROS) production, and impaired mitochondrial biogenesis and dynamics contribute to an energetic crisis and oxidative stress [14,15,16]. This compromised milieu is perpetuated by a vicious cycle involving chronic systemic inflammation and oxidative stress, which drive protein degradation via pathways such as the ubiquitin–proteasome system (UPS) and disrupt anabolic signaling [17,18,19]. More recently, epigenetic regulation has been identified as a novel and crucial layer of control, orchestrating these processes through alterations in histone deacetylases (HDACs) activity and microRNA (miRNA) expression profiles, thereby influencing muscle phenotype, inflammatory responses, and regenerative capacity in COPD [20,21,22,23,24,25]. Mechanical stress, inflammation, oxidative stress, and mitochondrial impairment are increasingly unified within a damage–regeneration–remodeling paradigm that offers a compelling mechanistic basis for the divergent clinical phenotypes of respiratory and limb muscles in COPD and provides a theoretical foundation for prioritizing the interconnected pathways discussed in this review [26].

This complex and interactive molecular landscape necessitates a refined conceptual framework that moves beyond the disuse model to view COPD-related SMD as a distinct myopathy with intrinsic disease drivers. Therefore, the purpose of this review is to synthesize recent advances by integrating current evidence on the roles of mitochondrial pathology, epigenetic reprogramming, and inflammatory pathways in driving muscle wasting and weakness. Furthermore, we will explore how deciphering this intricate network translates into the identification of novel circulating biomarkers for diagnosis and phenotyping and informs the development of targeted therapeutic strategies that extend beyond conventional exercise training. By connecting insights from bench to bedside, this review aims to provide a comprehensive overview that highlights the integrated nature of COPD-related SMD and charts a course for future research towards biomarker-guided phenotyping and personalized combinatorial management approaches. Throughout this review, we have explicitly stratified the evidence underpinning each mechanistic claim according to its hierarchical source. Findings derived from in vitro cellular models, murine in vivo experiments, and human observational or interventional studies are clearly annotated with their corresponding evidence tiers.

## 2. The Multifaceted Pathophysiology of COPD-Related Muscle Dysfunction

**Figure 1:**
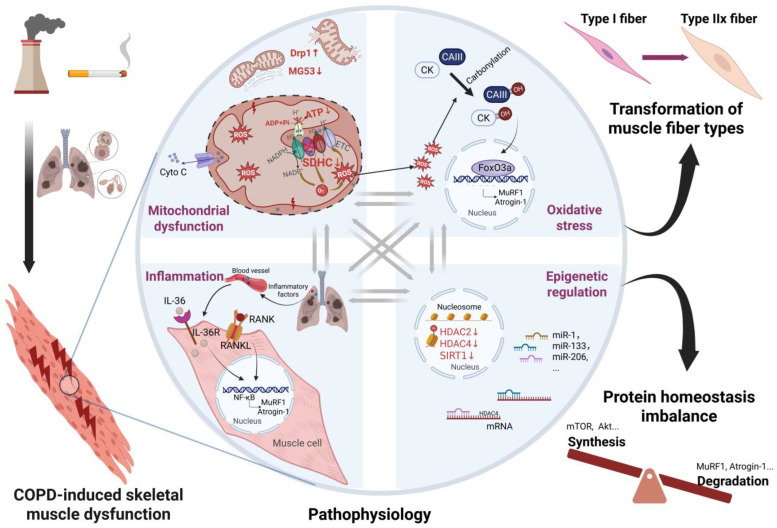
The multifaceted and interconnected pathophysiology of chronic obstructive pulmonary disease-induced skeletal muscle dysfunction. Skeletal muscle dysfunction (SMD) in chronic obstructive pulmonary disease (COPD) arises from a self-reinforcing network of pathological pathways, rather than a linear sequence. At its core, mitochondrial dysfunction drives a bioenergetic crisis characterized by impaired OXPHOS, succinate dehydrogenase subunit C (*SDHC*) deficiency, excessive fission mediated by high levels of dynamin-related protein 1 (Drp1) and low levels of mitsugumin 53 (MG53), and apoptosis initiated by mPTP opening and cytochrome c (Cyto c) release. This energetic deficit and associated reactive oxygen species (ROS) overproduction fuel a state of chronic oxidative stress, which promotes protein carbonylation and activates proteolytic signaling via the transcription factor FoxO3a. A pervasive catabolic drive is sustained by systemic inflammation originating from the lung and is amplified locally within the muscle by pathways such as the receptor activator of nuclear factor kappa-B ligand (RANKL)/RANK and IL-36/IL-36 receptor (IL-36R), leading to NF-κB-mediated upregulation of the ubiquitin-proteasomeubiquitin–proteasome system effectors muscle ring-finger protein-1 (MuRF1) and atrogin-1. Overseeing this maladaptive remodeling, epigenetic dysregulation orchestrates the transcriptional landscape through muscle-specific alterations in histone deacetylases (HDACs) activity and microRNA expression, which includes key myomiRs. This impairs regeneration and locks the muscle into an atrophic state. These four core pathways engage in extensive crosstalk, as indicated by bidirectional arrows, and collectively converge to disrupt protein homeostasis by tipping the balance towards degradation and to induce a fiber-type shift from oxidative type I to glycolytic type IIx fibers, ultimately manifesting as muscle atrophy, weakness, and fatigue (Created in BioRender. Gao, Q. (2026) https://BioRender.com/mbjcj5h).

The skeletal muscle wasting and weakness observed in COPD are not a mere consequence of physical inactivity but rather the endpoint of a complex interplay of multiple pathological pathways. Moving beyond the simplistic disuse model, this section delineates the intricate network of intrinsic disease processes that converge to disrupt muscle homeostasis. This intrinsic myopathy is driven by a core disruption in energy metabolism, perpetuated by oxidative and inflammatory insults, and masterfully orchestrated by profound epigenetic alterations, ultimately leading to disrupted cellular homeostasis and the clinical syndrome of SMD (Figure 1).

The selection of mitochondrial dysfunction, oxidative stress, inflammation, and epigenetic dysregulation as the core pathological network is theoretically grounded in their hierarchical and causal relationships, recently consolidated within the damage–regeneration–remodeling paradigm [26]. Mitochondrial dysfunction constitutes the primary energetic crisis, from which excessive ROS originate to fuel oxidative stress and activate NF-κB-mediated inflammatory signaling, ultimately driving UPS-dependent proteolysis. Epigenetic reprogramming operates as a higher-order regulatory layer that sustains this pro-atrophic transcriptional program and renders the muscle refractory to standard rehabilitation. Within this framework, satellite cell dysfunction and neuromuscular junction (NMJ) instability are positioned as parallel or downstream contributors, as NMJ degeneration is directly triggered by CS exposure, and satellite cell differentiation defects are partly mediated by HDAC9 upregulation, both of which are already encompassed within the inflammatory and epigenetic domains of our model [27,28,29].

To rigorously frame COPD-induced SMD as a distinct myopathy rather than non-specific atrophy, we propose defining hallmarks that establish its theoretical boundaries. First, the myopathy possesses an intrinsic mitochondrial OXPHOS defect, driven by aryl hydrocarbon receptor (*AHR*) overactivation, succinate dehydrogenase subunit C (*SDHC*) deficiency and uncoupled respiration. This defect persists even when physical activity levels are matched to healthy controls, indicating a disease-inherent bioenergetic failure beyond deconditioning [10,15]. Second, it exhibits muscle–type–specific epigenetic reprogramming with opposing HDAC expression patterns, namely HDAC4 upregulation in the chronically loaded diaphragm versus HDAC2/3/4 downregulation in the disused quadriceps and a globally hyperacetylated chromatin state in limb muscles that correlates with functional decline [24,30]. Third, a disease-stage-dependent myomiR signature comprising miR-1, miR-133a, and miR-206 is evident, wherein mild COPD elicits compensatory upregulation, whereas advanced disease shifts toward maladaptive overexpression that silences anabolic targets and promotes catabolic signaling [21,22,30]. Fourth, NMJ instability, evidenced by elevated circulating C-terminal agrin fragment (CAF22) and reduced neurotrophic support, acts as an upstream driver of denervation–reinnervation cycles, while HDAC9-mediated suppression of satellite cell differentiation impairs regenerative capacity [27,28,29]. Fifth, transcriptomic analyses reveal a failure to coordinately activate tissue remodeling and bioenergetics pathways, a transcriptional decoupling absent in simple disuse that directly links loss of oxidative phenotype to upregulation of ubiquitin–proteasome degradation genes [19,31]. Finally, these derangements coalesce into a blunted adaptive response to exercise training, characterized by impaired upregulation of PGC-1α, TFAM, and mitochondrial complexes, preventing complete functional restoration despite standard rehabilitation [10].

Collectively, these factors establish COPD-related SMD as a pathologically distinct entity, wherein the convergence of mitochondrial dysfunction, epigenetically locked inflammation, impaired regeneration, and NMJ instability creates a self-sustaining, refractory atrophic state fundamentally distinct from the reversible deconditioning observed in simple disuse.

### 2.1. Mitochondrial Dysfunction: The Core Energetic Crisis

Mitochondrial dysfunction is a cornerstone of skeletal muscle impairment in COPD, driving a core energetic crisis that underlies exercise intolerance, fatigue, and muscle wasting. Compelling evidence confirms these defects are intrinsic to the disease pathology, extending beyond mere disuse [10,16,32,33]. This multifaceted dysfunction encompasses bioenergetic failure, structural decay, and dysregulated dynamics, collectively compromising cellular energy homeostasis and contractile function.

#### 2.1.1. Bioenergetic Failure and Inefficient Compensation

A primary defect is the profound impairment of mitochondrial OXPHOS. Murine models of chronic CS exposure or genetic emphysema consistently show markedly reduced skeletal muscle oxygen consumption [34]. These findings are corroborated by human studies demonstrating impaired mitochondrial respiration in vastus lateralis biopsies of COPD patients [10]. This deficit is mechanistically linked to the sustained activation of *AHR*, with chronic smoke exposure shown to reduce mitochondrial OXPHOS by approximately 50% in a murine model. The causal role of *AHR* is underscored by the finding that muscle-specific *AHR* deletion in male mice attenuated smoke-induced OXPHOS impairment, while constitutive *AHR* activation in healthy mice recapitulated the defect [15]. While these murine data are compelling, translation to human COPD requires caution given species differences in *AHR* signaling and the complex interplay of comorbidities in patients. This *AHR*-driven pathology exhibits sexual dimorphism, highlighting complex, sex-dependent regulatory mechanisms.

The bioenergetic deficit is further exacerbated by specific enzymatic deficiencies within the mitochondrial matrix. A pivotal finding from an IL-13-driven emphysema model in mice is the downregulation of *SDHC*, a key component of both the tricarboxylic acid cycle and Complex II of the electron transport chain [14,34]. This downregulation leads to reduced succinate dehydrogenase (SDH) activity and succinate accumulation, indicating intrinsic enzyme dysfunction. The functional consequence of *SDHC* loss was confirmed in vitro via siRNA knockdown, which reduced cellular oxygen consumption. Crucially, the reversibility of this defect was demonstrated by in vivo *SDHC* overexpression in emphysematous mice, which restored SDH activity, normalized succinate levels, improved oxygen consumption, and enhanced muscle fatigue resistance, positioning *SDHC* as a key regulatory node and promising therapeutic target [14].

In response to this persistent energetic deficit, skeletal muscle initiates a compensatory increase in mitochondrial biogenesis, indicated by elevated citrate synthase activity [15,35]. However, this response is fundamentally inadequate, resulting in a state of inefficient compensation where increased mitochondrial quantity fails to rectify qualitative functional defects. This is evidenced in patients by a reduced ATP/O ratio (indicating uncoupled OXPHOS) and elevated nonphosphorylating respiration, despite normal levels of respiratory complexes [10]. While citrate synthase activity, a proxy for mitochondrial content, is elevated in COPD muscle, this quantitative increase fails to rectify functional deficits [15,35]. The newly synthesized mitochondria harbor qualitative defects, including impaired electron transport chain complex activity and uncoupled respiration, as evidenced by reduced ATP/O ratios and elevated nonphosphorylating respiration despite normal complex levels [10]. Critically, specific enzymatic deficiencies such as *SDHC* downregulation persist irrespective of mitochondrial mass [14]. Network-level disruption further compounds the issue, as excessive Drp1-mediated fission that leads to fragmentation compromises the functional integrity of the mitochondrial network regardless of total content. Moreover, mitochondrial quality control is globally dysregulated, with impaired coordination among biogenesis, dynamics, and mitophagy. Thus, the muscle’s compensatory increase in mitochondrial quantity represents a fundamentally inadequate response to a qualitative crisis. The resulting dissociation between oxygen consumption and ATP synthesis directly compromises the muscle’s ability to meet energetic demands during contraction.

#### 2.1.2. Structural Decay and Dysregulated Dynamics

The bioenergetic failure is compounded by pronounced structural and functional alterations in mitochondria. In patients with COPD, mitochondria from limb and respiratory muscles exhibit accelerated permeability transition pore (mPTP) kinetics and enhanced cytochrome c release, indicating a heightened susceptibility to apoptosis that correlates with reduced aerobic capacity and muscle mass [16]. Furthermore, a critical imbalance in mitochondrial fission and fusion dynamics contributes to the pathology. A shift towards excessive fission, driven by upregulation of Drp1, results in mitochondrial fragmentation, impaired OXPHOS, and increased ROS production. This process is promoted by CS extract-induced upregulation of myostatin (Mstn) in vitro [36]. The myokine mitsugumin 53 (MG53) emerges as a key regulator, which preserves membrane integrity and inhibits BCL2L13-mediated fission. MG53 deficiency in COPD exacerbates muscle atrophy, while supplementation with recombinant human MG53 rescued mitochondrial morphology and function, highlighting its therapeutic potential [7].

#### 2.1.3. Integration with Broader Pathophysiology

Mitochondrial dysfunction is not an isolated defect but actively initiates and perpetuates the cycle of muscle impairment through several key mechanisms. Beyond its direct impact on energy supply, manifesting as premature fatigue, transcriptomic analyses reveal a direct molecular link between loss of the oxidative phenotype and activation of atrophic pathways. The characteristic fiber-type shift is mechanistically coupled with upregulation of ubiquitin–proteasome degradation genes such as *FBXO32*/atrogin-1, directly tying mitochondrial inefficiency to proteolysis [31,35]. Finally, mitochondrial adaptability is severely constrained in COPD. Even when exercise training partially improves OXPHOS efficiency, the muscle cannot fully activate the complete transcriptional program that defines the adaptive response in healthy individuals, including upregulation of PGC-1α, TFAM, and mitochondrial respiratory complexes. This failure to mount a full adaptive response reveals a fundamental impairment in mitochondrial plasticity that critically limits the efficacy of standard rehabilitation [10].

Within the damage–regeneration–remodeling framework, emerging evidence has further dissected the interplay between mitochondrial stress and muscle regenerative capacity. Mitochondrial dysfunction not only compromises energy production during the energetically demanding process of muscle repair but also directly impairs the regenerative competence of muscle stem cells [37,38]. In COPD, the regenerative potential of skeletal muscle is markedly diminished, with mitochondrial metabolic dysfunction playing an independent and critical role in this inadequate myogenic regeneration [38]. Concurrently, systemic inflammation exerts additional suppressive effects on muscle repair and regeneration [39]. Consistent with these mechanistic insights, clinical observations demonstrate that although satellite cell activation and increased internal nuclei, indicators of attempted regeneration, are present in the vastus lateralis of severe COPD patients, the overall regenerative capacity remains profoundly compromised, particularly in those with sarcopenia [11]. Thus, rather than acting in isolation, mitochondrial dysfunction and inflammation converge to suppress the muscle’s intrinsic repair mechanisms, locking the tissue in a state of failed regeneration and progressive wasting.

Mitochondrial dysfunction in COPD skeletal muscle is a central pathophysiological feature characterized by defective OXPHOS, specific enzymatic deficiencies, structural fragmentation, and heightened apoptotic susceptibility. This core energetic crisis, which the muscle attempts to counter through incomplete compensatory mechanisms, not only directly impairs contractile function but also actively promotes fiber-type switching and catabolic signaling, thereby acting as a primary driver of the broader muscle pathology.

### 2.2. Oxidative Stress: A Perpetuating Insult

Intimately linked to and often originating from mitochondrial defects, oxidative stress represents a fundamental and self-perpetuating pathological insult in COPD-induced SMD. It arises from a chronic imbalance between the production of ROS and the capacity of endogenous antioxidant defense systems [40,41]. In COPD, this redox imbalance is exacerbated by multiple factors, including systemic inflammation, chronic hypoxemia, and direct exposure to CS, which collectively drive excessive ROS generation [42,43,44]. While low levels of ROS are essential for physiological signaling and muscle contraction, for example, through their role in excitation–contraction coupling, redox modulation of ryanodine receptors, and regulation of force production, their pathological accumulation leads to macromolecular damage, functional impairment, and the perpetuation of a vicious cycle of muscle wasting [43,45].

The consequences of sustained oxidative stress are multifaceted. Excessive ROS induces oxidative modifications of key cellular components, including lipids, DNA, and proteins. A study in human vastus lateralis biopsies identified that critical muscle proteins, such as creatine kinase (CK) and carbonic anhydrase III (CAIII), are specific targets for protein carbonylation, an irreversible modification that often leads to loss of function. In the vastus lateralis of COPD patients, the carbonylation of CK, a central enzyme in cellular energy buffering, is inversely correlated with both lung function and maximal oxygen consumption, directly linking oxidative damage to impaired muscle bioenergetics and exercise intolerance [18]. This oxidative environment also activates major proteolytic pathways. Mechanistically, oxidative stress promotes the dephosphorylation and nuclear translocation of the transcription factor FoxO3a, leading to the upregulation of atrophy-related genes muscle ring-finger protein-1 (MuRF1) and atrogin-1, thereby stimulating protein degradation via the UPS [42]. Furthermore, ROS can activate the NF-κB pathway, which in turn upregulates pro-inflammatory cytokines, creating a synergistic catabolic drive with inflammation [46]. Mitochondrial dysfunction is both a major source and a critical target of oxidative stress, creating a feed-forward cycle of damage. In murine models, CS exposure impairs mitochondrial biogenesis and function, as evidenced by decreased expression of PGC-1α and key mitochondrial markers such as Tom20 and COX2 [42]. In addition to mitochondrial sources, the activation of NADPH oxidase (Nox) systems is a significant contributor to the oxidative burden. The protective effects of the Nox inhibitor apocynin in CS-induced muscle atrophy in mice underscore the central role of Nox-derived ROS, which likely act by impairing the insulin-like growth factor-1 (IGF-1)/mTOR anabolic signaling pathway [19]. Recent evidence also highlights the role of oxidative stress in remodeling muscle fiber-type composition. Transcriptomic analyses of vastus lateralis biopsies from COPD patients reveal that the transition of muscle fibers to glycolytic type IIx/IIax fibers is associated with upregulated expression of oxidative stress-responsive genes [19]. This shift compromises the muscle’s oxidative capacity and perpetuates a cycle of metabolic inefficiency and further ROS overproduction.

The relationship between the oxidative-to-glycolytic fiber-type shift and the activation of atrophic pathways is complex and likely involves both direct transcriptional links and parallel responses to common upstream stressors. Direct evidence for transcriptional coupling comes from transcriptomic analyses showing co-expression networks that link loss of oxidative phenotype with upregulation of UPS-mediated degradation genes such as *FBXO32*/atrogin-1 [31]. The serum response factor (SRF)/miR-1 axis provides a mechanistic bridge in this context. Its downregulation simultaneously affects fiber-type specification through myocardin-related transcription factor (MRTF)-mediated control of slow-twitch gene expression and atrophy signaling via derepression of HDAC4 and suppression of IGF-1 [47]. Additionally, HIF-1α-driven metabolic reprogramming toward glycolysis has been implicated in both fiber-type shifting and sarcopenia. However, these processes may also occur independently as parallel consequences of shared stressors, as NMJ degeneration triggered by CS exposure drives recurring denervation–reinnervation cycles that result in both fiber-type grouping and atrophy [27,28]. Thus, while direct transcriptional links exist, the fiber-type shift and atrophy likely represent partially convergent outputs of a multifaceted pathological network.

Oxidative stress acts as a core perpetuating factor in COPD skeletal muscle by promoting direct macromolecular damage, fueling proteolysis, inducing mitochondrial dysfunction, and driving a maladaptive fiber-type shift. Therapeutic strategies aimed at restoring redox balance represent a crucial avenue for breaking this vicious cycle and preserving muscle mass and function in COPD.

### 2.3. Systemic and Local Inflammation: The Catabolic Drive

COPD is characterized by a state of persistent, low-grade systemic inflammation, often described as a “spill-over” from the pulmonary compartment. This inflammatory milieu is a central driver of extrapulmonary manifestations, with SMD being a critical consequence [40,48,49]. The systemic circulation in COPD patients exhibits elevated levels of pro-inflammatory cytokines, including TNF-α, IL-6, and IL-1β, which create a pervasive catabolic environment that disrupts skeletal muscle homeostasis [40,50]. Clinically, this systemic inflammatory state correlates strongly with decreased muscle strength, reduced exercise capacity, and diminished endurance [40]. The lung, upon chronic CS exposure, acts as a primary source of these inflammatory mediators. Recent research has highlighted novel signaling axes in this process. For instance, the IL-36/IL-36 receptor (IL-36R) pathway is significantly upregulated in response to CS and serves as a potent amplifier of the systemic inflammatory cascade. Genetic ablation of IL-36R in mice has been shown to attenuate not only lung inflammation and parenchymal destruction but also the associated SMD, underscoring its role as a key transducer of inflammatory signals from the lung to the muscle [51]. Beyond the systemic influx, a local inflammatory microenvironment is established within the skeletal muscle itself. Myofibers upregulate the expression of various cytokines and their receptors. A pivotal local pathway is the receptor activator of nuclear factor kappa-B ligand (RANKL)/RANK system, which is upregulated in the muscle of CS-exposed mice. Activation of RANKL/RANK signaling promotes a pro-catabolic state within the muscle by activating the master inflammatory regulator, NF-κB [52]. The convergence of systemic inflammatory mediators and the activation of local pathways like RANKL/RANK and IL-36/IL-36R creates a potent, self-sustaining catabolic drive.

The molecular mechanisms through which inflammation drives muscle catabolism are multifaceted, with the activation of protein degradation pathways being paramount. Inflammatory cytokines, particularly TNF-α, activate the NF-κB pathway, which in turn transcriptionally upregulates the expression of muscle-specific E3 ubiquitin ligases, MuRF1/*TRIM63* and atrogin-1/*FBXO32* [40,51]. These ligases are central executors of the UPS, targeting key structural and functional proteins for degradation. The critical link between inflammation and the UPS is evidenced by findings that RANKL neutralization suppresses CS-induced upregulation of MuRF1 and atrogin-1 in mice and that IL-36 cytokines directly upregulate *FBXO32* and *TRIM63* in human myotubes via NF-κB p65 activation [51,52]. Clinical studies corroborate these mechanisms, showing that elevated serum levels of inflammatory factors, including TNF-α and the potent myokine Mstn, are negatively correlated with quadriceps muscle strength in stable COPD patients [17]. Furthermore, systemic inflammation and oxidative stress form a vicious cycle that exacerbates muscle damage. Inflammatory factors can dysregulate antioxidant defenses, leading to ROS accumulation. The efficacy of the lipoprotein-associated phospholipase A2 (Lp-PLA2) inhibitor darapladib in mitigating CS-induced muscle dysfunction in a murine model highlights this interconnection, as its treatment concurrently reduced inflammatory cytokines, suppressed NF-κB activation, and rescued antioxidant Nrf2/HO-1 signaling [50]. This catabolic state is often compounded by comorbidities such as malnutrition, with the multiple-parameter malnutrition index being significantly higher in COPD patients and negatively correlated with muscle strength, suggesting an interplay that amplifies the catabolic drive [17].

In summary, a self-perpetuating cycle exists in COPD: pulmonary inflammation drives systemic inflammation, which instigates and amplifies local inflammation within skeletal muscle. This inflammatory milieu, mediated by key signaling pathways such as RANKL/RANK, IL-36/IL-36R, and NF-κB, forcefully drives muscle catabolism primarily through the transcriptional upregulation of atrogenes and activation of the UPS. The interplay between inflammation, oxidative stress, and malnutrition establishes systemic and local inflammation as a central therapeutic target in managing COPD-related myopathy.

### 2.4. Epigenetic Regulation: The Emerging Orchestrator

The persistent and coordinated alterations in gene expression across the aforementioned pathways suggest the involvement of a higher level of regulation. Epigenetic mechanisms provide this critical layer, explaining the sustained molecular imprint of COPD on skeletal muscle beyond the genetic code. They act as the orchestrator, fine-tuning the transcriptional programs of inflammation, metabolism, and regeneration in response to chronic stressors like CS and inflammation.

The focus on epigenetic regulation advances the field beyond traditional metabolic and inflammatory models in several fundamental ways. First, epigenetics provides a mechanistic explanation for the persistence of muscle dysfunction: histone modifications and miRNA signatures constitute a form of “molecular memory” that sustains pro-atrophic and pro-inflammatory gene expression even after the removal of inciting stimuli such as CS. Second, epigenetic mechanisms bridge environmental exposure and persistent transcriptional change, linking the chronic insult of tobacco smoke to enduring alterations in muscle phenotype. Third, this framework illuminates new therapeutic entry points, including HDAC9, HDAC2, and myomiRs, that are distinct from traditional anti-inflammatory or antioxidant approaches and may reverse the self-sustaining atrophic state [20,21,29,30]. Fourth, epigenetic dysregulation helps explain the blunted response to exercise training observed in COPD patients, suggesting that the muscle’s transcriptional machinery is epigenetically silenced; conventional rehabilitation may be insufficient to reactivate the full adaptive program [10]. Thus, the epigenetic lens not only deepens our mechanistic understanding but also opens novel therapeutic avenues and reframes the limitations of current standard-of-care interventions.

#### 2.4.1. Histone Modification

Histone modification, particularly through reversible acetylation and deacetylation, has emerged as a critical epigenetic mechanism fine-tuning gene expression in skeletal muscle without altering the DNA sequence [53,54]. This dynamic process, governed by the antagonistic actions of histone acetyltransferases and HDACs, directly influences chromatin architecture and transcriptional activity [55,56]. In COPD-induced SMD, distinct alterations in this equilibrium have been identified as key molecular determinants of muscle phenotype plasticity, atrophy, and functional impairment [24,30,57,58].

The functional consequences of histone acetylation are context-dependent: hyperacetylation typically relaxes chromatin to facilitate gene transcription, whereas hypoacetylation is associated with transcriptional repression. In patients with COPD, the pattern of histone modification exhibits significant variation depending on the muscle type, disease stage, and nutritional status. For instance, in the diaphragm of patients with mild-to-severe COPD and preserved body composition, HDAC4 protein levels are significantly upregulated, potentially representing an adaptive response to the chronic inspiratory load, while global acetylation markers remain unchanged [24]. Conversely, the limb muscles, such as the vastus lateralis in patients with advanced COPD and overt muscle weakness, display a marked shift towards hyperacetylation. This is evidenced by elevated levels of total lysine-acetylated proteins and acetylated histone H3, concomitant with reduced levels of key deacetylases, including HDAC3, HDAC4, and SIRT1. The roles of specific HDACs in COPD muscle dysfunction are complex and context-dependent, as summarized in Table 1. These alterations are correlated with decreased muscle strength and fat-free mass index, suggesting that a hyperacetylated state may promote a catabolic environment, potentially activating ubiquitin–proteasome pathways and disrupting protein homeostasis to accelerate muscle wasting [30].

Among the specific HDACs, HDAC2 has been extensively implicated in COPD-related muscle pathology. A consistent reduction in HDAC2 expression is observed in the quadriceps of COPD patients and correlates with disease severity and muscle weakness [23]. Mechanistically, HDAC2 downregulation leads to hyperacetylation and subsequent activation of the pro-inflammatory transcription factor NF-κB, driving the expression of inflammatory cytokines like TNF-α and IL-8, which contribute to muscle atrophy and apoptosis [20,23]. This pathway presents a therapeutic target, as evidenced in a murine model where theophylline attenuated muscle inflammation by upregulating HDAC2 and suppressing NF-κB [20]. Other HDAC isoforms also contribute to the complex pathology. HDAC5 protein levels are similarly reduced in COPD skeletal muscle and correlate with lung function impairment [23]. More recently, HDAC9 has been identified as a novel regulator of muscle regeneration. Chronic CS exposure upregulates HDAC9 expression in both murine myoblasts and skeletal muscle, impairing myogenic differentiation and myotube formation. Inhibition of HDAC9, either genetically or pharmacologically with TMP269, ameliorates smoke-induced muscle atrophy in mice and enhances satellite cell differentiation, partly through modulation of the AKT/mTOR and P53/P21 signaling axes [29]. Emerging evidence further reveals that upstream regulators of HDAC2 stability, such as USP47, can stabilize HDAC2 to suppress CS-induced skeletal muscle atrophy through the CYP1A1/ROS-mediated autophagy pathway, unveiling novel epigenetic regulatory nodes for therapeutic intervention [59].

The complexity of epigenetic regulation is further underscored by the interplay between HDACs and other regulatory layers, such as miRNAs. For example, the downregulation of the SRF/miR-1 axis in the quadriceps of COPD patients is associated with altered expression of myocardin-related transcription factors and changes in muscle fiber composition, indicating that histone-modifying enzymes operate within coordinated networks to fine-tune the muscle transcriptome in response to chronic stressors [47].

Histone modification represents a central and multifaceted epigenetic mechanism in COPD-induced SMD. The disease-stage and muscle-specific alterations in HDAC activity and acetylation status not only drive inflammation and impair regeneration but also unveil promising therapeutic targets for restoring muscle mass and function in COPD patients.

#### 2.4.2. miRNAs as Key Regulators and Biomarkers

Beyond histone modifications, miRNAs, small, non-coding RNA molecules, have emerged as pivotal epigenetic regulators of gene expression at the post-transcriptional level [60,61]. By binding to target mRNAs and inducing their degradation or translational repression, miRNAs fine-tune critical cellular processes, including muscle development, differentiation, fiber-type specification, and metabolic adaptation [62,63,64]. Among them, muscle-specific miRNAs, known as myomiRs, including miR-1, miR-133a/b, miR-206 and miR-499, are critically implicated in the pathogenesis of COPD-related SMD.

The dysregulation of myomiRs in COPD exhibits a complex, muscle-specific pattern, reflecting distinct adaptive and maladaptive responses in different muscle beds. In the diaphragm, which endures chronic inspiratory loading, a consistent downregulation of key myomiRs, including miR-1, miR-133a, and miR-206, has been observed [24,62]. This downregulation is hypothesized to be an adaptive epigenetic mechanism, potentially relieving the repression of growth-related pathways such as IGF-1 and SRF to promote a more fatigue-resistant phenotype in the face of sustained overload [24]. In stark contrast, the limb muscles, particularly the quadriceps, display a more dynamic and disease-stage-dependent miRNA signature. In mild COPD, an upregulation of miR-1 may serve a compensatory role, with its levels positively correlating with lung function and quadriceps strength [22]. However, as the disease progresses to advanced stages with pronounced muscle atrophy and weakness, this pattern shifts. Studies report a significant upregulation of miR-1, miR-206, and miR-27a, which is associated with the downregulation of target proteins like HDAC4 and IGF-1, disrupting the balance of muscle growth regulators and favoring catabolic processes and impaired differentiation over proliferation [30,47]. This maladaptive response in the limb muscles is further complicated by the involvement of non-myomiRs. For instance, miR-145-5p, elevated in the serum of COPD patients with muscle atrophy, has been shown to promote myotube apoptosis by inhibiting the pro-survival PI3K/Akt/mTOR pathway, highlighting a novel mechanism contributing to muscle loss [65]. The complex, muscle-specific expression patterns of these key miRNAs are systematically summarized in Table 2.

The distinct alterations in miRNA expression, coupled with their stability in biofluids, have positioned them as promising circulating biomarkers for muscle dysfunction in COPD. The detection of muscle-specific miRNAs in plasma offers a minimally invasive window into muscle status. Elevated plasma levels of miR-1, miR-133, miR-206, and miR-499 have been consistently reported in COPD patients, with specific miRNAs showing significant correlations with clinical parameters such as fat-free mass index, handgrip strength, and functional capacity [21,66]. miR-1 levels often inversely correlate with muscle mass, while miR-499 is associated with preserved type I fibers and better exercise performance [21]. The diagnostic potential is further enhanced by the discovery of extracellular vesicle-encapsulated myomiR signatures, such as a triple signature of miR-206, miR-133a-5p, and miR-133a-3p, which demonstrates high specificity in identifying patient subgroups with significant comorbidities [67]. Moreover, strong correlations between circulating miRNAs, such as miR-21 and miR-206, and established markers of inflammation, oxidative stress, and muscle damage reinforce their role in the integrated pathophysiology of COPD myopathy [66].

Therapeutically, miRNA networks are responsive to intervention. Studies in animal models have demonstrated that neuromuscular electrical stimulation can reverse the pathological upregulation of miR-1 and miR-133a, subsequently restoring fiber-type composition and reactivating pro-survival signaling via p-AKT, HDAC4, and SRF [68]. This suggests that modulating specific miRNA expression, such as enhancing miR-1 to promote myogenesis or inhibiting miR-145-5p to attenuate apoptosis, represents a promising frontier for targeted epigenetic therapies.

miRNAs serve a dual role in COPD-induced SMD: as master intracellular regulators of muscle plasticity across different disease stages and muscle types and as novel, non-invasive biomarkers with significant diagnostic, prognostic, and potential therapeutic value. A deeper understanding of their specific targets and regulatory networks is pivotal for developing miRNA-based strategies and integrating them into personalized management approaches for COPD patients.

### 2.5. Altered Protein Homeostasis: Synthesis and Degradation

The convergent endpoint of the energetic crisis, oxidative stress, inflammatory drive, and epigenetic reprogramming is the profound disruption of protein homeostasis, also known as proteostasis. This equilibrium is severely compromised in COPD, leading to accelerated muscle wasting and dysfunction. Multiple molecular pathways converge to dysregulate proteostasis, with the UPS and autophagy–lysosome pathway playing central roles.

The UPS is a major proteolytic system implicated in muscle atrophy. Key E3 ubiquitin ligases, such as MuRF1 and atrogin-1, are consistently upregulated in COPD models. In a CS-induced murine model of SMD, administration of the Lp-PLA2 inhibitor darapladib significantly attenuated the expression of MuRF1 and atrogin-1, concomitant with improved muscle mass and grip strength [50]. This suggests that inflammatory signaling, potentially via NF-κB activation, drives UPS-mediated proteolysis in COPD. Mitochondrial dysfunction and oxidative stress further exacerbate protein degradation. Impaired mitochondrial biogenesis and enhanced mitophagy, as observed in COPD rats and CS extract-treated L6 myotubes, are associated with elevated expression of autophagy-related proteins such as LC3B, ULK1, PINK1, and Parkin. The AMPK pathway appears to regulate this process, as its activation by the Bufei Jianpi formula suppressed mitophagy and restored mitochondrial function, thereby attenuating muscle atrophy [69].

Beyond degradation, impaired protein synthesis also contributes to muscle loss. Pathways involving mTOR, a critical regulator of anabolic processes, are often downregulated in COPD. In both clinical and preclinical settings, reduced phosphorylation of mTOR and its downstream targets has been linked to diminished muscle protein synthesis [69]. Additionally, systemic inflammation and elevated cytokines such as TNF-α, TWEAK, and Mstn correlate negatively with quadriceps strength and endurance in stable COPD patients [17]. These factors may inhibit synthesis pathways while simultaneously activating catabolic programs.

Importantly, the interplay between different proteostatic mechanisms is complex and often synergistic. For instance, oxidative stress can concurrently activate UPS and impair mitochondrial integrity, creating a vicious cycle of proteolysis and bioenergetic failure. Epigenetic mechanisms, including miRNA-mediated regulation of atrogenes and synthesis factors, may further fine-tune these processes, though this remains an emerging area of study.

Altered protein homeostasis in COPD skeletal muscle is characterized by a catabolic shift driven by inflammatory, oxidative, and metabolic stressors. Therapeutic strategies that simultaneously target protein degradation and synthesis, such as Lp-PLA2 inhibition or AMPK pathway modulation, hold promise for restoring muscle mass and function in this debilitating condition.

### 2.6. Other Contributing Mechanisms

Beyond the well-established pathways of mitochondrial dysfunction, oxidative stress, inflammation, and epigenetic regulation, the pathophysiology of COPD-induced SMD is further complicated by several other interconnected mechanisms. These include NMJ instability, gut-derived systemic inflammation due to increased intestinal permeability, and the action of specific circulating proteins such as dickkopf-related protein 3 (DKK3) and extracellular heat shock protein 72 [27,28,66,70]. These factors collectively exacerbate muscle wasting and weakness, representing additional molecular determinants of the disease. Genetic variation, particularly polymorphisms in the *FTO* and *AC090771.2* genes, has recently been associated with the heterogeneous severity of sarcopenia in COPD. Mechanistically, *FTO* depletion in mouse myotubes induces a senescent phenotype that is exacerbated by hypoxia, a common condition in COPD. These observations suggest that genetic factors interact with environmental stressors, such as chronic hypoxemia, to modulate muscle outcomes in a gene-by-environment manner [71].

## 3. Diagnostic and Prognostic Biomarkers

The accurate diagnosis and prognostic stratification of COPD-related SMD are critical for implementing timely interventions and improving patient outcomes. While traditional functional and morphological assessments remain the clinical cornerstone, there is a growing pursuit of objective, molecular biomarkers that can detect SMD at an earlier stage, reflect underlying pathological processes, predict disease progression, and predict response to therapy [72]. The ultimate goal is to move from a reactive to a proactive and personalized management strategy.

### 3.1. Conventional Functional and Morphological Assessments

Conventional assessments of SMD in COPD, encompassing functional and morphological evaluations, constitute the clinical and research cornerstone for diagnosing and monitoring the condition. These methods provide critical insights into the phenotypic manifestations of muscle impairment and serve as essential endpoints in therapeutic trials.

Functional assessments primarily focus on muscle strength and endurance. The quadriceps femoris is a key muscle for evaluation due to its vital role in locomotion and pronounced susceptibility in COPD. Strength is commonly measured via maximum voluntary contraction (MVC), with tests spanning isometric, isotonic, and isokinetic modalities. A systematic review indicated that resistance training significantly improved muscle strength, with a notably large effect size for isotonic strength, suggesting its heightened sensitivity to detect functional improvements post-intervention [73]. Complementing strength, endurance time during sustained submaximal contractions provides valuable information on fatigue resistance, which is characteristically impaired in patients with COPD [17].

Morphological assessments quantitatively evaluate muscle mass and structure. Common parameters include body mass index (BMI), fat-free mass index (FFMI), and the cross-sectional area (CSA) of major muscle groups like the quadriceps. While pooled analyses found no consistent exercise-induced improvements in BMI, significant benefits emerged in subgroup analyses restricted to high-quality studies, underscoring the impact of methodological rigor [73]. Furthermore, a study demonstrated significantly reduced femoral muscle volume and mid-thigh CSA in COPD patients versus healthy controls, reinforcing the link between muscle atrophy and functional decline [17].

Beyond pure strength and mass measurements, electrophysiological techniques serve as an extension of functional assessment. Surface electromyography, in particular, offers a window into neuromuscular efficiency. Patients with COPD demonstrate decreased root-mean-square values of surface electromyography in the vastus lateralis, rectus femoris, and vastus medialis during quadriceps MVC, indicating impaired muscle activation capacity [17].

Notwithstanding their utility, these conventional approaches have limitations. Significant heterogeneity in testing protocols can hinder cross-study comparisons [73]. Moreover, results can be confounded by factors such as age, disease severity, and nutritional status, necessitating careful contextual interpretation. Most importantly, these measures largely capture the downstream consequences of muscle pathology rather than the specific molecular drivers, limiting their utility for targeted therapeutic decision-making.

Conventional functional and morphological assessments provide a robust, clinically relevant framework for evaluating SMD in COPD. While they remain indispensable for diagnosis and monitoring, their limitations highlight the need for protocol standardization and, crucially, their integration with novel molecular and imaging biomarkers for a more comprehensive and mechanistic understanding of SMD.

### 3.2. Novel Circulating Biomarkers

The integration of conventional assessments with novel circulating biomarkers holds the key to a more precise and early diagnosis of SMD in COPD. Moving beyond the limitations of physical performance tests and imaging, circulating biomarkers offer a unique window into the underlying molecular pathophysiology, enabling non-invasive detection, risk stratification, phenotyping, and monitoring of therapeutic responses. Recent research has identified a diverse array of promising biomarkers reflective of key pathological processes, including NMJ instability, epigenetic dysregulation, systemic inflammation, metabolic derangements, and altered protein homeostasis (Table 3). These biomarkers effectively bridge the gap between the mechanistic pathways and clinical application.

#### 3.2.1. Neuromuscular and Epigenetic Regulators

The stability of the NMJ and the epigenetic control of gene expression are critical for muscle health, and their disruption is a key feature of COPD myopathy. The C-terminal agrin fragment (CAF)22, a byproduct of agrin cleavage, has emerged as a sensitive and extensively validated serum marker of NMJ instability. Elevated CAF22 levels in COPD patients are consistently and inversely correlated with handgrip strength and appendicular skeletal muscle mass index and demonstrate dynamic associations with physical performance metrics such as gait speed [27,28]. The diagnostic power of CAF22 is enhanced when combined with other NMJ-related factors, such as brain-derived neurotrophic factor (BDNF) and glial cell line-derived neurotrophic factor (GDNF), which are significantly reduced in COPD. A biomarker panel comprising CAF22, BDNF, and GDNF has been shown to yield superior diagnostic accuracy for sarcopenia compared to any single marker alone [28]. Notably, patients with asthma-COPD overlap exhibit a higher degree of NMJ degradation than either disease alone, underscoring the need for phenotype-specific biomarker interpretation [74].

In parallel, myomiRs have gained prominence as stable, circulating epigenetic regulators. These miRNAs, often encapsulated within extracellular vesicles, provide a snapshot of muscle-specific gene expression. A distinctive “triple signature” of miR-206, miR-133a-5p, and miR-133a-3p was significantly upregulated in extracellular vesicles from GOLD group B patients, demonstrating potential for patient stratification [67]. Other studies have corroborated the alteration of myomiRs, including miR-1, miR-133, miR-206, and miR-499, in COPD plasma, with levels correlating with muscle strength, mass, and systemic inflammation [21,66]. Notably, the dysregulation of these myomiRs is linked to impaired signaling pathways within muscle, such as the SRF/MRTF axis and the expression of key genes like HDAC4 and IGF-1, underscoring their role in epigenetic remodeling of muscle phenotype [30,47].

#### 3.2.2. Systemic Inflammatory, Metabolic, and Catabolic Mediators

Beyond the NMJ and epigenetic regulation, systemic factors reflecting inflammation, metabolic stress, and activated catabolic pathways contribute significantly to muscle wasting. Growth differentiation factor-15 (GDF-15), a stress-responsive cytokine, is markedly elevated during acute exacerbations and is inversely correlated with muscle CSA. Its reduction following targeted interventions like early standardized enteral nutrition underscores its role as a dynamic marker of acute catabolic stress [75,76]. The gut–muscle axis has also been implicated, with zonulin, a marker of intestinal permeability, being linked to muscle decline. Elevated plasma zonulin levels are associated with increased systemic inflammation and oxidative stress, and their reduction following pulmonary rehabilitation correlates with improved muscle strength and function [70]. Furthermore, molecules involved in specific signaling pathways offer mechanistic insights. DKK3, a modulator of Wnt signaling, is overexpressed in COPD patients with sarcopenia. It promotes mitochondrial dysfunction and myotube atrophy via interaction with the cytoskeleton-associated protein 4 receptor and demonstrates strong diagnostic performance for predicting dynapenia and sarcopenia [77]. Conversely, the tripeptide glycine–histidine–lysine with Cu (GHK-Cu), which possesses antioxidative and anti-inflammatory properties, is reduced in COPD plasma. Its levels correlate positively with muscle mass and negatively with TNF-α, while exogenous administration attenuates muscle atrophy via SIRT1 activation in a murine model, highlighting its dual role as a biomarker and therapeutic agent [42]. Lp-PLA2, an enzyme involved in inflammatory lipid metabolism, is another circulating marker negatively associated with muscle mass and function, and its inhibition ameliorates muscle wasting in preclinical models [50]. More recently, calprotectin, a damage-associated molecular pattern abundantly secreted by neutrophils and macrophages, has emerged as a dual-purpose biomarker reflecting both systemic inflammation and skeletal muscle catabolism in COPD. Serum calprotectin levels were significantly elevated in COPD patients with SMD compared to those without and negatively correlated with handgrip strength, quadriceps strength, and rectus femoris thickness. Notably, serum calprotectin could effectively predict sarcopenia in two independent COPD cohorts [78]. Emerging evidence also suggests that elevated plasma glucagon-like peptide-1 (GLP-1) levels are independently associated with sarcopenia in elderly COPD patients, with a linear inverse relationship between GLP-1 and skeletal muscle index, expanding the repertoire of potential circulating biomarkers [79].

#### 3.2.3. Emerging Myokines and Muscle-Derived Factors

The exploration of muscle-derived cytokines (myokines) and other muscle-specific proteins has revealed new candidates that serve as direct reporters of muscle status. MG53 was recently identified as a muscle-specific myokine with diagnostic and therapeutic relevance. Plasma MG53 levels are significantly reduced in COPD patients with sarcopenia and correlate positively with muscle strength and mass. Functionally, MG53 deficiency exacerbates CS-induced mitochondrial dysfunction, while its supplementation restores mitochondrial integrity and alleviates atrophy, positioning it as a highly promising biomarker [7]. In the context of vascular dysregulation, angiopoietin-2 is upregulated in the muscle and plasma of COPD patients, with levels correlating with disease severity and muscle wasting, suggesting its role in impaired muscle perfusion [80].

**Table 3:** Novel circulating biomarkers associated with SMD in COPD.

Category	Biomarker(s)	Primary Pathophysiological Role	Correlation with Muscle Parameters	Evidence Source	Key References
Neuromuscular and epigenetic	CAF	NMJ instability, synaptotoxicity	Negative with muscle strength and mass	Human (serum)	[27,28]
	BDNF, GDNF	Neurotrophic support for NMJ integrity and axonal regeneration	Positive with muscle health; reduced in COPD	Human (serum)	[28]
	MyomiRs (e.g., miR-206, miR-133, miR-1)	Epigenetic regulation of muscle phenotype, regeneration, and inflammation	Altered levels correlate with disease severity, muscle mass, and strength	Human (plasma, serum, EVs)	[21,66,67]
Systemic and metabolic	GDF-15	Stress-responsive cytokine, mediator of catabolic stress	Negative with muscle CSA	Human (serum)	[75]
	Zonulin	Marker of intestinal permeability, gut–muscle axis mediator	Negative with muscle strength and function	Human (plasma)	[70]
	Lp-PLA2	Enzyme in inflammatory lipid metabolism	Negative with muscle mass and function	Human (plasma), murine model	[50]
	Calprotectin	Damage-associated molecular pattern; drives TLR4/RAGE-mediated inflammation, promotes proteolysis	Negative with muscle strength, mass, and physical function	Human (serum), murine model	[78]
	DKK3	Inhibitor of Wnt signaling, induces mitochondrial dysfunction	Negative with muscle strength, mass, and exercise capacity	Human (plasma), in vitro	[77]
	GHK-Cu	Tripeptide with antioxidative and anti-inflammatory properties	Positive with muscle mass and antioxidative capacity	Human (plasma), murine model	[42]
	GLP-1	A negative predictor and potential pathophysiological mediator of muscle wasting	Negative with muscle mass and function	Human (plasma)	[79]
Muscle-derived and angiogenic	MG53	Muscle-specific myokine, crucial for mitochondrial integrity and repair	Positive with muscle strength, mass, and physical performance	Human (plasma), murine model	[7]
	Angiopoietin-2	Marker of vascular dysregulation and impaired angiogenesis	Negative (upregulated in muscle wasting)	Human (muscle biopsies, plasma)	[80]

In summary, the landscape of circulating biomarkers for COPD-related muscle dysfunction is rapidly expanding, encompassing markers of NMJ integrity, epigenetic regulation, systemic stress, and specific signaling pathways. The future of biomarker application lies in their integration into multi-parameter panels. Combining these distinct but complementary markers will provide a holistic view of the individual’s pathophysiological state, facilitating not only early diagnosis and precise risk stratification but also guiding the selection of targeted therapeutic interventions in the era of precision medicine for COPD.

Despite the promise of biomarker-guided precision medicine for COPD-related SMD, several practical barriers must be acknowledged. On the technical and operational front, assay standardization poses a major hurdle: circulating miRNA quantification varies widely across platforms and laboratories, and extracellular vesicle processing adds further complexity to sample preparation [67]. Cost and accessibility further constrain the clinical deployment of multi-parameter biomarker panels in routine practice, particularly in resource-limited settings. On the validation front, most proposed biomarkers, including myomiRs, CAF22, DKK3, and calprotectin, have been evaluated only in cross-sectional studies, and prospective validation in longitudinal cohorts with predefined clinical endpoints remains largely lacking [7,27,50,77,78]. On the translational front, biomarker levels fluctuate during acute exacerbations, complicating their interpretation in the dynamic clinical course of COPD, and clinically actionable thresholds have not been established for most candidates, hindering the translation of biomarker signals into therapeutic decisions [75]. Overcoming these barriers will require coordinated efforts across disciplines to develop standardized protocols, validate biomarkers in prospective trials, and establish evidence-based algorithms for clinical decision-making.

### 3.3. Emerging Imaging Biomarkers: Shear Wave Elastography for Assessing Muscle Quality

While traditional imaging modalities like dual-energy X-ray absorptiometry and computed tomography are well-established for assessing muscle mass in sarcopenia and COPD, they offer limited insight into muscle quality, a critical determinant of functional status [81,82]. Emerging ultrasound-based techniques, particularly shear wave elastography (SWE), address this gap by providing a non-invasive means to quantify muscle stiffness as a surrogate for muscle quality [83,84,85,86]. This parameter offers a functional correlate to the molecular pathology described earlier. A study applied SWE to the rectus femoris muscle in COPD patients, revealing that the mean elasticity index (SWE_mean_) was significantly lower than in healthy controls and exhibited a progressive decline with advancing GOLD stages. Crucially, SWE_mean_ demonstrated excellent intra- and inter-observer reliability, supporting its clinical reproducibility. This parameter was independent of anthropometric variables like height, weight, and BMI, yet showed strong correlations with pivotal clinical outcomes, including pulmonary function, exercise capacity, muscle strength, and physical performance. Notably, these associations were consistently stronger than those observed with conventional grayscale ultrasound measures of muscle thickness (RF_thick_) or CSA (RF_csa_), underscoring SWE’s unique ability to capture functional properties beyond mere morphology [87]. Further strengthening its biological relevance, SWE_mean_ showed significant negative correlations with circulating biomarkers of muscle wasting and systemic inflammation, such as GDF-15, resistin, and TNF-α. This suggests that SWE-derived stiffness may reflect underlying inflammatory and metabolic disturbances in COPD, effectively bridging imaging findings with the molecular landscape. Most importantly, SWE_mean_ achieved superior diagnostic accuracy for sarcopenia compared to RF_thick_ and RF_csa_, highlighting its potential as a robust, non-invasive biomarker for the early detection and monitoring of muscle quality decline [87]. Recent studies have further validated SWE as a feasible tool to reflect lower limb dysfunction in COPD patients, reinforcing its clinical utility [88]. These findings position SWE as a promising tool that integrates real-time visualization with the assessment of muscle mechanical properties. Future research should validate its utility in longitudinal cohorts, during acute exacerbations, and in assessing responses to therapeutic interventions like pulmonary rehabilitation.

## 4. Therapeutic Interventions: From Rehabilitation to Targeted Therapy

Given the multifaceted pathophysiology of COPD-related SMD, therapeutic strategies must extend beyond simply improving lung function to directly target the peripheral muscle defects. The current management landscape spans exercise training as the cornerstone, nutritional and pharmacological support, and an emerging frontier of molecularly targeted therapies.

### 4.1. Exercise Training: The Cornerstone with Limitations

Exercise training is universally established as the cornerstone non-pharmacological intervention for managing SMD in COPD. Its efficacy in improving muscle mass, strength, exercise capacity, and health-related quality of life is well-documented [89,90]. Systematic reviews confirm that structured programs, encompassing endurance training, resistance training, and combined training, significantly enhance peripheral muscle strength and functional performance in stable COPD patients. The benefits are modality-specific: resistance training preferentially augments isotonic strength and lean mass, while endurance training leads to superior gains in endurance and peak aerobic capacity [73,91,92].

However, the adaptive response to exercise is neither uniform across all patients nor consistent across different muscle subsystems. A pivotal limitation lies in the modality-specific effects on mitochondrial pathology, a core feature of COPD. Conventional concentric endurance training effectively promotes mitochondrial biogenesis and respiratory capacity, evidenced by elevated PGC-1α expression and enhanced respiration. In contrast, eccentric ergometer training, despite enabling higher mechanical loads with reduced cardiometabolic stress, fails to induce comparable mitochondrial adaptations, potentially due to differences in the metabolic and molecular signaling pathways activated by the two modalities [12]. This divergence underscores a critical gap: modalities that effectively improve mass and mechanical function may not rectify the underlying mitochondrial dysfunction. Furthermore, even when mitochondrial efficiency improves, patients with COPD exhibit a blunted adaptive response in maximal mitochondrial respiration and key biogenesis regulators like TFAM compared to healthy controls [10]. This suggests that intrinsic disease-related factors, including systemic inflammation, oxidative stress, and epigenetic alterations, constrain mitochondrial plasticity.

This heterogeneity necessitates a personalized exercise prescription. Patients with profound muscle weakness may benefit from eccentric or resistance-oriented regimens to build functional strength. Notably, resistance training has demonstrated efficacy even during acute exacerbations by preventing declines in quadriceps force and promoting an anabolic shift in muscle gene expression, characterized by reduced Mstn and an elevated myogenin/MyoD ratio [93,94]. However, caution is warranted with eccentric loading, as it may induce greater muscle damage and enzyme leakage in COPD patients [95]. Conversely, patients with predominant exercise intolerance due to impaired oxidative metabolism may respond more favorably to high-intensity interval or endurance-focused training, which has recently been shown to alleviate COPD-induced SMD via the bromodomain-containing protein 4/PGC-1α axis, restoring mitochondrial function and oxidative fiber composition [96].

Adjunctive strategies, such as supplemental oxygen during exercise, can enhance adaptations. In patients with exercise-induced desaturation, oxygen supplementation has been associated with greater improvements in peak work rate and quadriceps CSA, mitigating hypoxia-mediated limitations in muscle remodeling [97]. A meta-analysis further supports that combining long-term oxygen therapy with exercise synergistically improves exercise endurance and quality of life [98]. Nevertheless, fundamental challenges persist. Molecular heterogeneity underlies the variable clinical response. Time-course studies indicate that, despite comparable gains in mass and strength, molecular anabolic and signaling responses are often attenuated in COPD patients compared to healthy controls. Moreover, post-exercise nutritional supplementation has not consistently augmented training-induced gains, indicating potential anabolic resistance [11].

Importantly, exercise training, while clinically beneficial in COPD, often fails to fully normalize molecular and functional parameters in muscle [10]. This limited efficacy may be partly attributable to an epigenetic constraint that restricts the muscle’s adaptive response. Emerging evidence indicates that exercise training modulates epigenetic marks such as histone acetylation and miRNA expression in skeletal muscle [55]. In the context of COPD, however, persistent dysregulation of HDACs and myomiRs may constrain the transcriptional response to exercise. For example, HDAC9 upregulation in CS-exposed muscle impairs satellite cell differentiation, thereby limiting the regenerative response to training-induced muscle damage [29]. Similarly, pathological elevation of miR-1 and miR-206 in advanced COPD suppresses key anabolic targets and blunts the hypertrophic response to resistance training [30]. Collectively, these epigenetic constraints help explain why exercise training, despite its established benefits, cannot completely reverse the molecular and functional deficits in COPD patients [10]. Future therapeutic strategies may therefore need to combine exercise with epigenetic modulators, such as HDAC inhibitors or miRNA-targeted agents, to overcome this epigenetic barrier.

While exercise training is indispensable for ameliorating muscle dysfunction in COPD, its benefits are partial and modality-dependent. Current paradigms are insufficient to fully reverse mitochondrial defects or consistently restore peak aerobic capacity in all individuals. Future approaches should consider integrating sequential or combined training modalities, for instance, employing initial eccentric ergometer training to build strength, followed by concentric endurance training to target oxidative capacity, with physiological support such as supplemental oxygen. Furthermore, the integration of targeted molecular interventions is crucial to overcome these limitations and achieve comprehensive functional restoration.

### 4.2. Nutritional and Pharmacological Strategies

Beyond the cornerstone of exercise training, nutritional and pharmacological interventions represent pivotal adjunctive strategies to counteract SMD in COPD [99,100]. These approaches aim to target the core pathological pathways detailed in previous sections, including mitochondrial dysfunction, oxidative stress, inflammation, and dysregulated protein homeostasis, thereby offering hope for restoring muscle mass and functional capacity (Table 4).

Nutritional support has emerged as a promising avenue to modulate the intrinsic muscle milieu. Several supplements have been investigated for their potential to enhance bioenergetics and mitigate catabolic drives. As an essential cofactor for mitochondrial fatty acid β-oxidation, L-carnitine addresses the metabolic derangement commonly observed in COPD. Patients frequently exhibit low serum carnitine levels, which correlate with disease severity. Supplementation facilitates efficient fuel utilization, reduces oxidative stress, and improves muscle bioenergetics. Clinically, administration of L-carnitine for six weeks reduced blood lactate levels, improved exercise tolerance, and enhanced respiratory muscle strength [101]. Its efficacy in reducing exacerbation frequency further supports its role as a safe and cost-effective metabolic enhancer [102]. The pivotal role of oxidative stress in promoting muscle fatigue has spurred interest in high-dose antioxidant therapy. A randomized controlled trial demonstrated that intravenous infusion of ascorbate/vitamin C significantly boosted systemic antioxidant capacity and attenuated the development of quadriceps fatigue during exercise in COPD patients [103]. This was associated with improved muscle perfusion and reduced ventilatory demand, highlighting the potential of antioxidants to protect muscle contractility from oxidant-mediated damage. Omega-3 long-chain polyunsaturated fatty acids (LC PUFAs) such as EPA and DHA exert potent anti-inflammatory effects by incorporating into cell membranes and suppressing pro-inflammatory eicosanoids. Supplementation has been shown to improve lean body mass and quadriceps strength [104]. Similarly, vitamin D deficiency, prevalent in COPD, is linked to muscle weakness and mitochondrial dysfunction. Repletion of vitamin D improves mitochondrial OXPHOS, enhances antioxidant defense, and promotes myogenic differentiation, leading to gains in muscle strength and exercise capacity [105,106].

Beyond its bronchodilatory effects, theophylline exhibits anti-inflammatory properties in skeletal muscle. In a murine model of emphysema, aminophylline treatment reduced levels of pro-inflammatory cytokines in the gastrocnemius muscle by upregulating HDAC2 and suppressing NF-κB activation [20]. This epigenetic mechanism underscores its potential to mitigate muscle inflammation, although its impact on mass restoration in short-term interventions appears limited. While the evidence for many nutritional and pharmacological strategies is compelling, their translation into clinical practice requires careful consideration. Some conventional agents, such as angiotensin-converting enzyme inhibitors, have shown limited efficacy in improving muscle function in isolation, highlighting that single-target approaches may be insufficient for established myopathy [109].

Several nutritional and pharmacological strategies discussed above operate in part through epigenetic mechanisms. Among dietary factors, vitamin D, beyond its classical role in calcium homeostasis, modulates epigenetic regulation via the vitamin D receptor, which recruits histone acetyltransferases and chromatin-remodeling complexes to target gene promoters. Vitamin D repletion has been shown to enhance mitochondrial OXPHOS and antioxidant defense through such pathways [105]. Omega-3 LC PUFAs influence the epigenetic landscape by modulating DNA methylation patterns and histone acetylation profiles, in addition to their direct anti-inflammatory effects [104]. Among pharmacological agents, the tripeptide GHK-Cu activates SIRT1, a NAD^+^-dependent deacetylase, thereby inhibiting FoxO3a-mediated proteolysis and enhancing mitochondrial biogenesis via PGC-1α deacetylation [42]. Theophylline exerts its anti-inflammatory effects in skeletal muscle through HDAC2 upregulation and subsequent NF-κB suppression [20]. The multi-targeted Bufei Jianpi formula, meanwhile, modulates mitochondrial function via the AMPK pathway, which intersects with epigenetic regulation through NAD^+^/SIRT1 signaling and PGC-1α activation, suggesting that its benefits may also involve epigenetic reprogramming of metabolic gene networks [69]. Collectively, many current interventions for COPD-induced SMD engage epigenetic mechanisms, and optimizing their efficacy may require consideration of the patient’s baseline epigenetic profile.

Notably, multi-targeted approaches, particularly those derived from traditional medicine, offer a holistic strategy. Traditional Chinese medicine formulations offer a holistic, multi-targeted approach, often demonstrating benefits across several pathological pathways simultaneously. The Bufei Jianpi formula has shown comprehensive benefits in improving skeletal muscle structure and function in COPD models. Bufei Jianpi formula administration enhances mitochondrial morphometry and function, increases ATP production, and reduces oxidative stress and apoptosis in both limb and respiratory muscles [107]. Mechanistically, the Bufei Jianpi formula improves mitochondrial membrane potential, inhibits mPTP opening, and suppresses caspase-3-mediated apoptosis [108]. Furthermore, its effects are mediated through the activation of the AMPK signaling pathway, leading to the upregulation of mitochondrial biogenesis markers, including PGC-1α, TFAM, and the suppression of mitophagy-related proteins like LC3B, PINK1, and Parkin [69]. These multi-faceted actions position the Bufei Jianpi formula as a potent complementary therapy for COPD-related myopathy.

### 4.3. Novel Targeted Agents and Future Directions

While conventional therapies such as exercise and nutritional support remain foundational in the management of COPD-related SMD, their efficacy is often limited by disease severity and patient adherence. Consequently, there is a growing impetus to develop targeted molecular therapies that address the specific pathophysiological pathways underlying muscle wasting. Recent preclinical and translational research has unveiled a spectrum of promising targets, spanning inflammatory signaling, proteolytic pathways, mitochondrial quality control, regulated cell death, and epigenetic regulation, heralding a new era of precision medicine in this domain (Table 5).

The pursuit of agents that directly counteract the chronic inflammatory and proteolytic drive in COPD has yielded significant insights. The IL-36/IL-36R axis has emerged as a key mediator of systemic inflammation and muscle atrophy. A study demonstrated that IL-36R antagonism attenuates CS-induced SMD in mice by suppressing the NF-κB p65 pathway and the subsequent expression of the E3 ubiquitin ligases *FBXO32*/Atrogin-1 and *TRIM63*/MuRF1 [51]. Similarly, targeting Lp-PLA2, an enzyme linked to systemic inflammation and oxidative stress, has shown promise. The Lp-PLA2 inhibitor darapladib ameliorated muscle wasting in a CS-exposed mouse model, reducing the expression of atrogin-1 and MuRF1 and suppressing NF-κB activation [50]. Another inflammatory mediator that has recently emerged as both a circulating biomarker and a druggable target is calprotectin. Using a CS-exposed mouse model, a recent study demonstrated that oral administration of paquinimod, a specific calprotectin inhibitor, significantly attenuated CS-induced muscle wasting. Mechanistically, paquinimod suppressed the CS-induced upregulation of atrogin-1 and MuRF1, reduced local pro-inflammatory cytokines, and restored the antioxidant Nrf2/HO-1 pathway [78]. These findings position IL-36R blockers, Lp-PLA2 inhibitors, and calprotectin inhibitors as novel anti-inflammatory and anti-proteolytic strategies.

Dysregulation of myokine signaling represents another therapeutic frontier. Research has elucidated an imbalance in the Mstn/irisin axis in CS-induced models, where upregulated Mstn promotes atrophy and suppresses the protective myokine irisin. Targeting this axis with Mstn-neutralizing antibodies or irisin mimetics could restore anabolic capacity [110]. Furthermore, the same group identified ferroptosis, an iron-dependent form of regulated cell death, as a critical mechanism [111]. They demonstrated that CS exposure induces ferroptosis in skeletal muscle via Mstn-mediated upregulation of HIF-2α in a murine model. Pharmacological inhibition of HIF-2α or the use of ferroptosis inhibitors like UAMC-3203 significantly preserved muscle mass and function, revealing a novel therapeutic avenue to combat myocyte loss.

Given the central role of mitochondrial dysfunction in COPD myopathy, strategies to enhance mitochondrial quality control are of paramount interest. The tripeptide GHK-Cu has demonstrated protective effects. GHK-Cu administration attenuates CS-induced muscle wasting by activating SIRT1, which in turn inhibits FoxO3a-mediated proteolysis, enhances mitochondrial biogenesis via PGC-1α, and bolsters antioxidant defenses through Nrf2 activation [42]. Another key player is the myokine MG53. MG53 deficiency exacerbates CS-induced atrophy by disrupting mitochondrial fission in a murine model, whereas supplementation with recombinant human MG53 rescued mitochondrial morphology and function by promoting the degradation of the pro-fission protein BCL2L13 [7]. Additionally, *AHR*, chronically activated by tobacco smoke, has been implicated in mitochondrial dysfunction. Skeletal muscle-specific *AHR* knockout ameliorated CS-induced mitochondrial deficits in male mice, suggesting *AHR* antagonists as a sex-specific therapeutic strategy [15].

Epigenetic regulators and specific signaling nodes offer precise targets for intervention. Evidence points to HDAC9 as a critical negative regulator of muscle regeneration. It was demonstrated that inhibition of HDAC9, either genetically or pharmacologically with TMP269, enhances satellite cell differentiation and improves muscle mass and strength in CS-exposed models by activating the AKT/mTOR pathway and suppressing the P53/P21 senescence axis [29]. Emerging data further indicate that HDAC6-mediated PHB2 degradation via acetylation–ubiquitination crosstalk drives mitophagy failure and muscle atrophy in COPD models, revealing additional HDAC family members as potential therapeutic targets [112]. Beyond epigenetics, efficacy has been shown for modulation of the nitric oxide/soluble guanylate cyclase (sGC)/cyclic guanosine monophosphate (cGMP) pathway. Specifically, the sGC stimulator BAY 41–2272 was found to attenuate limb muscle atrophy in a guinea pig model of chronic CS exposure, reducing proteolytic markers and restoring muscle fiber CSA [113].

At a systems level, co-expression networks enriched for genes involved in ubiquitination, insulin signaling, and muscle contraction have been identified through transcriptomic analyses and are associated with abnormal myofiber proportions in COPD patients. These networks underscore the complexity of muscle remodeling and suggest that multi-gene or pathway-based interventions may be necessary [19].

While the targeted agents discussed above hold considerable therapeutic promise, their clinical translation requires careful appraisal of potential systemic risks. *AHR*, for instance, is a pleiotropic transcription factor that regulates not only xenobiotic metabolism but also immune homeostasis, barrier function, and developmental processes. Chronic *AHR* antagonism could impair physiological detoxification and immune surveillance [15]. Lp-PLA2 inhibitors, despite showing efficacy in preclinical models, have yielded mixed results in large cardiovascular outcome trials, underscoring the need for careful patient selection and rigorous safety monitoring [50]. HDAC inhibitors, including HDAC9 antagonists and emerging HDAC6-targeted strategies, face the challenge of isoform selectivity, given the diverse and sometimes opposing functions of different HDAC family members across tissues [29,112]. Ferroptosis inhibitors lack established predictive biomarkers, and their long-term effects on iron homeostasis and redox balance remain unknown. MuRF1 inhibition requires careful evaluation of potential off-target effects, given the ubiquitin–proteasome system’s broader role in cellular homeostasis. Collectively, these considerations indicate that the path from preclinical promise to clinical practice demands not only efficacy validation but also thorough pharmacokinetic, toxicological, and pharmacovigilance studies in relevant patient populations. A biomarker-guided stratification approach, as advocated in this review, may help identify patients most likely to benefit while minimizing exposure of those at greatest risk of off-target effects.

**Figure 2:**
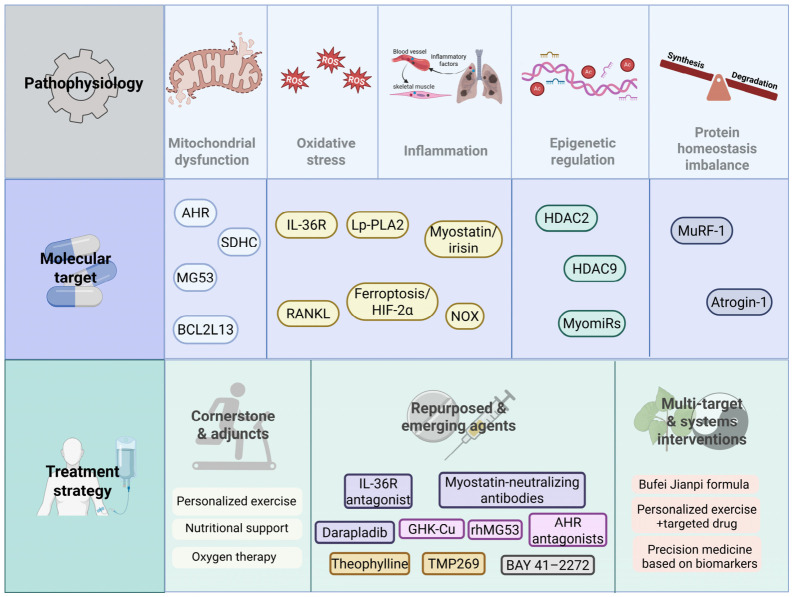
A translational roadmap for targeting chronic obstructive pulmonary disease-induced skeletal muscle dysfunction: from pathophysiology to therapeutic avenues. This schematic integrates the molecular determinants of chronic obstructive pulmonary disease (COPD)-induced skeletal muscle dysfunction (SMD) with current and emerging therapeutic strategies, outlining a translational pipeline from bench to bedside. The core pathological mechanisms inform the identification of specific molecular targets, which are the focus of a stratified therapeutic arsenal. This arsenal is conceptualized in three tiers: (1) Cornerstone and adjuncts, comprising foundational interventions like personalized exercise training, nutritional support, and supplemental oxygen; (2) Repurposed and emerging agents, including molecularly targeted therapies such as Lp-PLA2 inhibitors (darapladib), IL-36R antagonists, myostatin-neutralizing antibodies, *AHR* antagonists, HDAC9 inhibitors (TMP269), rhMG53, and ferroptosis inhibitors; and (3) Multi-target and systems interventions, encompassing holistic approaches like the Bufei Jianpi formula, rational combination therapies such as exercise plus targeted drugs, and the future paradigm of biomarker-guided precision phenotyping. The ultimate goal is to move beyond a one-size-fits-all approach towards personalized management tailored to an individual’s dominant pathophysiological drivers (Created in BioRender. Gao, Q. (2026) https://BioRender.com/6301x53).

The evolving landscape of targeted therapy for COPD-induced muscle dysfunction is rapidly expanding beyond conventional approaches. By focusing on specific molecular nodes within inflammatory, proteolytic, mitochondrial, cell death, and epigenetic pathways, these emerging strategies offer tangible hope for reversing or halting the progression of sarcopenia and significantly improving the functional status and quality of life for patients with COPD. The ultimate challenge and opportunity lie in the rational integration of these targeted agents with foundational exercise and nutritional strategies, guided by the biomarker-based phenotyping, to deliver truly personalized and effective management for COPD-induced SMD. The multitude of interventions, from the cornerstone of exercise training through nutritional and pharmacological adjuncts to the burgeoning field of molecularly targeted agents, highlights the vast potential for managing COPD-induced SMD. This integrated therapeutic landscape, bridging foundational approaches with precision medicine, is summarized in Figure 2.

## 5. Conclusions and Future Perspectives

SMD in COPD is a prevalent and debilitating comorbidity that transcends the simplistic notion of disuse atrophy. A substantial body of evidence now firmly establishes it as a distinct disease-specific myopathy, driven by an intricate and self-reinforcing interplay of pathological mechanisms. The core of this pathology is a mitochondrial energetic crisis, characterized by defective OXPHOS, aberrant dynamics, and impaired biogenesis and plasticity. This crisis is perpetuated by chronic oxidative stress and systemic inflammation, which collectively activate proteolytic systems such as the UPS and suppress anabolic signaling, thereby tipping the metabolic balance towards net protein loss. Overseeing and entrenching these processes is a layer of epigenetic dysregulation, involving altered activities of histone deacetylases, including HDAC2 and HDAC9, and miRNAs, which orchestrates a persistent pro-atrophic and pro-inflammatory gene expression signature in the COPD muscle. The emergence of novel biomarkers, ranging from circulating myomiRs and proteins such as CAF22 and DKK3 to functional imaging assessments like SWE, provides powerful non-invasive tools for diagnosing, phenotyping, and monitoring this complex condition.

While exercise training remains the indispensable cornerstone of management, its inability to fully reverse the underlying molecular defects underscores the imperative for adjunctive and targeted therapies. Promisingly, pre-clinical studies have delineated a spectrum of potential molecular targets, including *AHR*, Lp-PLA2, IL-36R, and the restorative GHK-Cu and MG53 axis, heralding a new era of mechanism-based interventions.

Looking ahead, several key avenues warrant focused exploration to advance the field towards precision medicine and translate these mechanistic insights into clinical practice. First, future research must move beyond a one-size-fits-all approach through the validation and implementation of integrated biomarker panels. Combining circulating miRNAs such as miR-206 and miR-133, NMJ markers such as CAF22, and atrophy-related proteins such as DKK3 will be crucial to stratify patients into distinct molecular phenotypes. This stratification will enable truly personalized therapeutic strategies, matching interventions to an individual’s dominant pathological drivers; for example, prescribing specific antioxidants for a high oxidative stress phenotype or neurotrophic support for severe NMJ degradation. Second, the development of rational combinatorial and synergistic strategies is paramount. Combining interventions with complementary mechanisms, for instance, by concurrently pairing exercise training with pharmacological agents like GHK-Cu or an Lp-PLA2 inhibitor to simultaneously enhance mitochondrial biogenesis and curb inflammation, may yield synergistic effects superior to any single modality. Third, a deeper mechanistic understanding is needed in specific areas, particularly in elucidating sex-specific molecular responses, which will inform tailored interventions. Furthermore, exploring whether initiating multi-faceted therapy during mild or even pre-COPD stages can prevent or delay the onset of irreversible muscle wasting represents a crucial paradigm shift from treatment to prevention and early intervention.

In summary, the conceptualization of COPD-induced SMD has evolved from a passive complication to an active therapeutic target. By integrating insights from molecular pathology, leveraging novel biomarkers for patient stratification, and embracing a personalized and combinatorial treatment philosophy, the future of managing this devastating aspect of COPD is poised to become significantly more effective and patient-centric. The journey from bench to bedside will be guided by this integrated framework, ultimately aiming to preserve muscle health, functional independence, and quality of life for individuals with COPD.

## Figures and Tables

**Table 1:** Roles of specific HDACs in COPD-induced SMD.

HDAC Isoform	Change in COPD Muscle	Proposed Mechanisms and Consequences	Clinical/Therapeutic Correlation	Evidence Source
HDAC2	Downregulated	Loss of repression on NF-κB, leading to its hyperacetylation and activation.	Correlates with disease severity and muscle weakness.	Human (quadriceps biopsies)
		Increased transcription of pro-inflammatory cytokines (TNF-α, IL-8).	Theophylline exerts anti-inflammatory effects via HDAC2 upregulation.	Murine model
		Promotes inflammation-driven atrophy and apoptosis.	/	Murine model, human correlational
HDAC3	Downregulated	Contributes to the overall hyperacetylated state in limb muscle.	Associated with advanced disease and muscle wasting.	Human (vastus lateralis)
		May disrupt normal protein turnover and energy metabolism.		Human
HDAC4	Upregulated in diaphragm (mild-severe COPD)	Diaphragm: Upregulation may be an adaptive response to chronic loading.	Represents muscle-specific and disease-stage-specific regulation.	Human (diaphragm)
	Downregulated in limb muscle (severe COPD)	Limb Muscle: Downregulation contributes to global hyperacetylation, potentially activating catabolic pathways.	Correlates with muscle strength and fat-free mass index.	Human (vastus lateralis)
HDAC5	Downregulated	Specific mechanistic role in muscle is less defined but implicated in transcriptional repression.	Correlates with the degree of lung function impairment.	Human (quadriceps)
HDAC9	Upregulated	Impairs myogenic differentiation and myotube formation.	Pharmacological inhibition ameliorates muscle atrophy and enhances regeneration in experimental models.	Murine myoblasts, in vivo murine model
		Suppresses satellite cell-mediated regeneration.		Murine model
		Acts via inhibition of AKT/mTOR and activation of P53/P21 signaling.		Murine model
SIRT1	Downregulated	As a NAD^+^-dependent deacetylase, its loss may link metabolic stress to epigenetic dysregulation and impaired mitochondrial function.	Associated with muscle weakness and cachexia.	Human (vastus lateralis)

**Table 2:** Key miRNAs in COPD-induced SMD.

miRNA	Source of Samples	Expression Pattern in COPD	Major Targets	Functional Consequences	Clinical Implications/Biomarker Potential
miR-1	Human (diaphragm)	Consistently downregulated	HDAC4, IGF-1, MRTFs	Regulates myogenesis, differentiation, and fiber-type switching (slow-twitch maintenance). Downregulation may promote growth pathways; upregulation linked to maladaptive catabolism.	Circulating levels inversely correlate with fat-free mass index and correlate with forced expiratory volume in 1 s and quadriceps force. A potential biomarker for muscle mass and function.
	Human (quadriceps)	Mild COPD: Upregulated (potentially compensatory)			
		Advanced COPD: Conflicting reports			
miR-133a	Human (diaphragm)	Downregulated	SRF (involved in myocyte proliferation)	Balances myoblast proliferation and differentiation.	Circulating levels may be downregulated. Part of an EV-encapsulated “triple signature” (with miR-206) for identifying patient phenotypes.
	Human (quadriceps)	Upregulated in advanced COPD			
miR-206	Human (diaphragm)	Downregulated	HDAC4, IGF-1, Connexin 43	Promotes muscle differentiation and regeneration; chronic upregulation may drive atrophy.	Circulating levels are elevated in severe COPD with dysfunction and correlate with handgrip strength, CRP, and oxidative stress markers.
	Human (quadriceps)	Upregulated in advanced COPD			
miR-499	Human (limb muscle)	Altered in limb muscle, associated with fiber-type shift	SOX6	Promotes and maintains type I (slow-twitch) muscle fibers.	Circulating levels correlate with preserved type I fiber proportion and better exercise performance. A potential biomarker for favorable fiber-type status.
miR-145-5p	Human (serum), in vitro (C2C12 myotubes)	Elevated in COPD patients with muscle atrophy	PI3K/Akt/mTOR pathway	Inhibits cell survival signaling, promotes myotube apoptosis, and exacerbates muscle wasting.	A novel circulating biomarker and therapeutic target specifically linked to apoptotic pathways in COPD muscle atrophy.

**Table 4:** Overview of nutritional and pharmacological strategies for counteracting SMD in COPD.

Specific Agent/Class	Primary Molecular Targets and Mechanisms	Key Demonstrated Outcomes	Evidence Source	Key References
L-carnitine	Fatty acid transport into mitochondria; Enhances β-oxidation; Reduces oxidative stress	Improved exercise tolerance, reduced blood lactate, enhanced respiratory muscle strength, reduced exacerbations	Human RCT	[101,102]
Antioxidants (Vitamin C)	Systemic antioxidant capacity; Mitigates oxidant-mediated contractile impairment and improves perfusion	Attenuated quadriceps fatigue during exercise, improved femoral vascular conductance	Human RCT	[103]
Omega-3 long-chain polyunsaturated fatty acids (LC PUFAs) (EPA and DHA)	Cell membrane composition; Suppresses pro-inflammatory eicosanoids and cytokines	Increased lean body mass, improved skeletal muscle mass and quadriceps strength	Human RCT/meta-analysis	[104]
Vitamin D	Vitamin D receptor (VDR); Enhances mitochondrial OXPHOS and antioxidant defense; Promotes myogenic differentiation	Improved muscle strength, exercise capacity, and mitochondrial function	Human observational, murine model	[105,106]
Theophylline/aminophylline	Epigenetic regulation (upregulates HDAC2); Suppresses NF-κB-mediated inflammation	Reduced muscle levels of IL-8 and TNF-α in preclinical models	Murine model	[20]
Bufei Jianpi formula	AMPK signaling pathway; Enhances mitochondrial biogenesis (PGC-1α), suppresses mitophagy (PINK1/Parkin), inhibits apoptosis	Improved mitochondrial function and morphometry, increased ATP production, reduced apoptosis in limb and respiratory muscles	Murine/rat models	[69,107,108]

**Table 5:** Summary of novel targeted agents for COPD-induced SMD.

Category	Therapeutic Target	Representative Agent(s)	Key Mechanism of Action	Research Stage	Evidence Source	Reference
Inflammatory and proteolytic signaling	IL-36R	IL-36R antagonist (theoretical)	Inhibits NF-κB pathway, downregulates E3 ubiquitin ligases (*FBXO32*, *TRIM63*)	Preclinical	Murine model	[42]
	Lp-PLA2	Darapladib	Reduces oxidative stress and NF-κB activation, suppresses atrogin-1/MuRF1	Preclinical	Murine model	[41]
	Calprotectin	Paquinimod	Prevents calprotectin from binding TLR4/RAGE; suppresses NF-κB activation, downregulates atrogin-1/MuRF1, reduces oxidative stress and pro-inflammatory cytokines	Preclinical (biomarker validated in human cohorts)	Murine model, human serum validation	[71]
Myokine network and cell death	Mstn	Mstn-neutralizing antibodies	Blocks atrophic signaling, restores anabolic capacity	Preclinical	Murine model	[100]
	Irisin	Irisin mimetics	Compensates for suppressed irisin, promotes muscle protection	Preclinical	Murine model	[100]
	Ferroptosis/HIF-2α	HIF-2α inhibitors, UAMC-3203	Inhibits iron-dependent cell death, reduces lipid peroxidation	Preclinical	Murine model	[101]
Mitochondrial quality control	SIRT1 activation	GHK-Cu	Enhances mitochondrial biogenesis (via PGC-1α), boosts antioxidant defenses (via Nrf2), inhibits proteolysis	Preclinical	Murine model	[34]
	MG53	Recombinant human MG53	Stabilizes mitochondrial membranes, promotes degradation of fission protein BCL2L13	Preclinical	Murine model, human plasma	[7]
	*AHR*	*AHR* antagonists	Ameliorates mitochondrial dysfunction, improves OXPHOS	Preclinical	Murine model	[15]
Epigenetic and signaling modulation	HDAC9	TMP269 (HDAC9 inhibitor)	Promotes satellite cell differentiation via AKT/mTOR, suppresses cellular senescence	Preclinical	Murine model	[51]
	Soluble guanylate cyclase (sGC)	BAY 41–2272 (sGC stimulator)	Elevates cyclic guanosine monophosphate (cGMP) levels, attenuates proteolysis and muscle atrophy	Preclinical	Guinea pig model	[102]

## Data Availability

No new data were created or analyzed in this study. Data sharing is not applicable to this article.

## Abbreviations

The following abbreviations are used in this manuscript:

*AHR*	Aryl hydrocarbon receptor
BDNF	Brain-derived neurotrophic factor
BMI	Body mass index
CAF	C-terminal agrin fragment
CAIII	Carbonic anhydrase III
CK	Creatine kinase
cGMP	Cyclic guanosine monophosphate
COPD	Chronic obstructive pulmonary disease
CSA	Cross-sectional area
CS	Cigarette smoke
DKK3	Dickkopf-related protein 3
FFMI	Fat-free mass index
GDF-15	Growth differentiation factor-15
GDNF	Glial cell line-derived neurotrophic factor
GHK-Cu	Glycine–histidine–lysine with Cu
GLP-1	Glucagon-like peptide-1
HDACs	Histone deacetylases
IGF-1	Insulin-like growth factor-1
IL-36R	IL-36 receptor
LC PUFAs	Long-chain polyunsaturated fatty acids
Lp-PLA2	Lipoprotein-associated phospholipase A2
MG53	Mitsugumin 53
miRNA/miR	MicroRNA
mPTP	Mitochondrial permeability transition pore
MRTF	Myocardin-related transcription factor
Mstn	Myostatin
MVC	Maximum voluntary contraction
MuRF1	Muscle ring-finger protein-1
NMJ	Neuromuscular junction
Nox	NADPH oxidase
OXPHOS	Oxidative phosphorylation
RANK	Receptor activator of nuclear factor kappa-B
RANKL	Receptor activator of nuclear factor kappa-B ligand
ROS	Reactive oxygen species
SDH	Succinate dehydrogenase
*SDHC*	Succinate dehydrogenase subunit C
sGC	Soluble guanylate cyclase
SMD	Skeletal muscle dysfunction
SRF	Serum response factor
SWE	Shear wave elastography
UPS	Ubiquitin–proteasome system
